# Spinal Cord Infarction: A Single Center Experience and the Usefulness of Evoked Potential as an Early Diagnostic Tool

**DOI:** 10.3389/fneur.2020.563553

**Published:** 2020-10-27

**Authors:** Dougho Park, Byung Hee Kim, Sang Eok Lee, Ji Kang Park, Jae Man Cho, Heum Dai Kwon, Su Yun Lee

**Affiliations:** ^1^Department of Rehabilitation Medicine, Pohang Stroke and Spine Hospital, Pohang-si, South Korea; ^2^Department of Radiology, Pohang Stroke and Spine Hospital, Pohang-si, South Korea; ^3^Department of Neurosurgery, Pohang Stroke and Spine Hospital, Pohang-si, South Korea; ^4^Department of Neurology, Pohang Stroke and Spine Hospital, Pohang-si, South Korea

**Keywords:** spinal cord infarction, early diagnosis, motor evoked potential (MEP), somatosensory evoked potential (SEP), diffusion MRI, transverse myelitis

## Abstract

**Background:** Spinal cord infarction (SCI) is a rare disease and its early diagnosis is challenging. Here, we described the clinical features and imaging findings of SCI, and assessed the results of evoked potential (EP) studies to elucidate their diagnostic role in the early stage of SCI.

**Methods:** We retrospectively analyzed 14 patients who had spontaneous SCI. The demographic, neurological, and temporal profiles of the SCI patients were identified. We reviewed the imaging findings and assessed the changes in them over time. To review EP, central motor conduction time (CMCT) and somatosensory evoked potential (SEP) values were obtained. We also enrolled 15 patients with transverse myelitis (TM), and compared the clinical, radiological and electrophysiological features between SCI and TM patients.

**Results:** The ages of the SCI patients ranged from 54 to 73 years. Nine patients (64.3%) showed nadir deficits within 6 h. The most common type of clinical visit was via the emergency center. Nine patients (64.3%) presented with peri-onset focal pain. The median initial modified Rankin scale score was 3. For 9 patients (64.3%), initial T2 imaging findings were negative, but subsequent diffusion weighed imaging (DWI) showed diffusion restriction. Vertebral body infarction was observed in 5 patients (35.7%). EP data were available for 10 SCI patients. All 8 patients who had their CMCT measured showed abnormalities. Among them, motor evoked potentials were not evoked in 6 patients at all. SEP was measured in 10 patients, and 9 of them showed abnormalities; one of them showed no SEP response. For 5 patients, the EP studies were done prior to DWI, and all the patients showed definite abnormalities. The abnormalities in the EP findings of the SCI patients were more profound than those of the TM patients, even though the duration from the onset to the start of the study was much shorter for SCI patients.

**Conclusion:** SCI can be diagnosed based on typical clinical manifestations and appropriate imaging studies. Our study also indicates that immediate sensory and motor EP study can have an adjuvant diagnostic role in the hyperacute stage of SCI, and can improve the accuracy of diagnosis.

## Introduction

Spinal cord infarction (SCI) is an uncommon cause of spinal cord injury, and has been reported to account for ~1.0% of all stroke cases (1, 2). SCI patients present with severe and diverse neurologic deficits depending on the anatomical location and the extent of the infarction. SCI patients generally experience an abrupt onset of motor weakness, loss of senses of pain and temperature, altered proprioception, and bowel and bladder dysfunctions. SCI has also been reported to have generally unfavorable outcomes (3, 4).

Early recognition of SCI immediately after the onset of symptoms is difficult; thus, SCI is considered a challenging disease (5). Differential diagnosis of SCI should exclude diseases such as acute inflammatory myelitis, multiple sclerosis, intra or extramedullary tumor, infectious conditions, and hematoma, which are characterized by features of spinal cord disorders; brain lesions and peripheral nerve lesions should be excluded as well (6). Transverse myelitis (TM) in particular should be excluded, as previous studies have reported that about 15% of patients diagnosed with TM were actually SCI patients (7, 8).

Although imaging has been an important tool for diagnosing SCI, studies have shown that early detection of SCI by imaging alone is difficult (9). Often, conventional magnetic resonance imaging (MRI) done after the onset of symptoms does not show any positive findings. Diffusion weighted imaging (DWI) is not generally performed together with the initial conventional MRI. Consequently, SCI is often masked in the hyperacute stage. Even if DWI is initially performed in cases of suspected SCI, it is possible that a diffusion MRI performed during the hyperacute stage will show false negative findings (10, 11). A standardized imaging protocol for the initial diagnosis of SCI has not been proposed yet. Thus, SCI is a disease with a high probability of delayed diagnosis or misdiagnosis in its early stage.

Evoked potential (EP) study has proven to be a useful diagnostic tool for evaluating the motor and sensory tracts of the spinal cord, and for diagnosing myelopathy (12–14). Central motor conduction time (CMCT) can mainly assess the corticospinal tract from the primary motor cortex to the spinal cord, and reflects the functions of not only the pyramidal tracts but the extrapyramidal tracts of the adjacent anterolateral columns (15). CMCT can be obtained by subtracting the peripheral motor conduction time from the onset latency of the motor evoked potential (MEP) induced by transcranial magnetic stimulation (16, 17). For the measurement of somatosensory evoked potential (SEP), stimuli originating from the peripheral nerve are mainly transmitted along the sensory pathway of the dorsal column of the spinal cord and the medial lemniscus of the brain stem, and are then finally recorded in the thalamus. SEP is a useful tool that takes the overall perfusion state into account, and is also useful for evaluating the function of the posterior column of the spine, which is difficult to detect using MEP alone (18, 19). Multimodal EP study, which utilizes both MEP and SEP simultaneously, is known to be a highly sensitive and specific testing method with a rapid response to abnormalities in the central neural pathway. However, research on how EP could play a distinct role in the early diagnosis of SCI is yet to be carried out.

In this study, we described the clinical features and the imaging findings of SCI patients admitted to our hospital. In addition, we analyzed the role of immediate sensory and motor EP study in the early diagnosis of SCI. We additionally elucidated the usefulness of EP study by comparing the EP results of TM patients with those of SCI patients, and we also identified the clinical and radiological differences between SCI and TM patients.

## Materials and Methods

### Study Design and Patient Inclusion

We conducted a retrospective observational study based on patient medical records, imaging findings, and electrodiagnostic test records. Out of the patients that visited our hospital from October 2011 to March 2020, we selected 16 patients who were diagnosed with spontaneous SCI. All patients underwent conventional spinal and brain MRI immediately after their visit according to their clinical symptoms. Following the MRI, patients suspected of SCI underwent spinal DWI and apparent diffusion coefficient (ADC) study. For SCI diagnosis, we utilized the diagnostic criteria proposed by Zalewski et al. (20) in 2019. Thereafter, we enrolled patients with definite or probable spontaneous SCI into our study. For this study, the diagnostic criteria for definite spontaneous SCI included: (1) no preceding myelopathy or traumatic lesion of the spinal cord; progression from onset to nadir within 12 h or less (onset to nadir); or severe deficits that rapidly developed within 12 h or less; (2) no spinal cord compression; (3) intramedullary high signal intensity (HSI) in T2 image; (4) one of the following: presence of diffusion restriction in DWI/ADC study, vertebral body infarction, arterial dissection, or occlusion of adjacent artery; and (5) differentiation from other similar diseases. The criteria for probable spontaneous SCI are similar to the aforementioned criteria; however, (4) is replaced with non-inflammatory findings of the CSF study. Two of the patients enrolled as SCI patients were excluded because they did not meet the criteria for definite or probable SCI. Thus, a total of 14 patients were finally selected for the study, and all of them were diagnosed with definite SCI.

To compare the clinical, radiological, and electrophysiological findings of SCI and TM patients, we enrolled 15 patients diagnosed with TM within the same sampling period. Our diagnostic criteria for inflammatory myelopathy were mainly based on the proposal released by the Transverse Myelitis Consortium Working Group in 2002 (21). The final diagnoses of both SCI and TM were made by experienced neurologists, neurosurgeons, and radiologists based on clinical features, imaging findings, and CSF study results.

### Observation Items

To identify the clinical features of SCI, we reviewed demographic factors and vascular risk factors such as hypertension, diabetes mellitus, hyperlipidemia, and smoking. For each patient, we checked the motor, sensory, and autonomic symptoms at nadir, and checked for peri-onset focal adjacent pain. To describe functions and outcomes, we used the modified Rankin scale (mRS). To identify the temporal profiles of the clinical features, we categorized the duration from onset of symptoms to nadir into the following four categories: <6, 6–24, 24–72, and 72 h or more. We also determined the type of visit and the duration from the onset of symptoms to the time of the hospital visit. Initial serum inflammatory indexed–white blood cell (WBC) count, erythrocyte sedimentation rate (ESR), and C-reactive protein (CRP) level were also verified.

To check the characteristics and findings of imaging studies, we investigated the duration from the onset of symptoms to the completion of the first spinal MRI study and the presence of HSI in the initial T2 image. Subsequently, we determined the duration from the onset of symptoms to the completion of the DWI/ADC study, the presence of diffusion restriction, and the involved spinal levels. We also investigated the presence of vertebral body infarction.

To check the EP study, we adopted the values of CMCTs recorded from the abductor pollicis brevis muscle (APB-CMCT) and the tibialis anterior muscle (TA-CMCT). We also used the value of median SEP and tibial SEP. For patients who had saddle symptoms or hypo-reflexic bowel and bladder, we also conducted a pudendal SEP. Based on previous reports, the cut-off value was set at 8.8 ms for APB-CMCT and 17.2 ms for TA-CMCT (22). The cut-off value for latencies of median SEP, tibial SEP, and pudendal SEP were 20.3, 41.3, and 41.1 ms, respectively (23). All patients who underwent the EP study also underwent a routine nerve conduction study (NCS) and electromyography (EMG) to rule out other peripheral neuropathies, polyneuropathy or polyradiculopathy. We also measured the duration from the onset of symptoms to the completion of the electrodiagnostic study. To compare the EP results of the SCI and TM patients, of the 15 TM patients, we selected 10 who underwent EP testing.

We expressed continuous variables as median values (interquartile range, IQR), and categorical variables were expressed as number of patients and percentage values. The Mann-Whitney test was used to compare SCI and TM group data.

## Results

### Clinical Features

A total of 14 SCI patients were enrolled in this study. The ages of the patients ranged from 54 to 73 years. There were 8 male patients (57.1%). Eleven patients (78.6%) had one or more vascular risk factors, and hypertension, which 6 patients presented with, was the most common. Eleven patients (78.6%) had autonomic dysfunctions. The median score of the initial mRS test was 3 (2,4), indicating a relatively profound neurologic deficit. We were able to conduct a follow-up test with the mRS for 10 patients. Among them, 4 patients scored 3 or higher, indicating that they had limited functional independency in their daily life (Table 1).

**Table 1:** Clinical features of all spinal cord infarction patients.

				**Symptoms at nadir**				
**Case**	**Age/sex**	**Vascular risk factors**	**Focal pain**	**Motor**	**Sensory**	**Bladder/bowel**	**mRS**	**fu mRS (fu time)**
1	54/F	None	Yes	Hemiparesis	Numbness, paresthesia	Absent	2	Unavailable
2	63/M	HL, CAD	Yes	Quadriparesis	Paresthesia	Yes	3	2 (4 m)
3	71/M	HTN	Absent	Paraparesis	Decreased pain sense, paresthesia	Absent	2	2 (12 m)
4	59/F	HTN	Yes	Monoplegia	Altered proprioception, loss of pain sense	Yes	4	Unavailable
5	71/M	HTN, CAD	Yes	Monoplegia	Altered proprioception, paresthesia	Yes	4	Unavailable
6	55/F	DM	Yes	Paraplegia	Paresthesia	Yes	4	3 (6 m)
7	73/F	none	Absent	Quadriplegia	Numbness, decreased pain sense	Yes	4	4 (3 m)
8	68/M	HTN, SM	Absent	Paraparesis	Paresthesia, decreased pain sense	Yes	3	2 (3 m)
9	60/F	HL	Yes	Paraparesis	Altered proprioception, loss of pain sense, paresthesia	Yes	3	1 (2 m)
10	63/M	none	Absent	Monoplegia	Numbness, paresthesia	Absent	2	1 (12 m)
11	54/M	SM	Absent	Paraparesis	Decreased pain sense, paresthesia	Yes	2	Unavailable
12	71/M	HTN	Yes	Paraparesis	Altered proprioception, paresthesia	Yes	2	1 (12 m)
13	71/F	HTN	Yes	Paraplegia	Loss of pain sense, paresthesia	Yes	4	3 (12 m)
14	68/M	DM	Yes	Paraplegia	Altered proprioception, loss of pain sense, paresthesia	Yes	4	3 (12 m)

*mRS, modified Rankin scale; fu, follow up; M, male; F, female; HL, hyperlipidemia; CAD, coronary artery disease; HTN, hypertension; DM, diabetes mellitus; SM, smoking*.

The temporal profiles of the SCI patients were as follows: regarding the duration from the onset of symptoms to nadir, 9 patients (64.3%) presented with the hyperacute type (<6 h), making it the most common type recorded; for the type of hospital visit, 10 patients (71.4%) visited the hospital via the emergency center; and for the duration from the onset to the hospital visit, the first visit was made within 4 h [0.17 day [0.10, 1.00]]. The time it took to obtain the first T2 image was 8 h (0.33 day [0.14, 1.00]), and the duration from the onset to the completion of the diffusion MRI study was 2.75 days (1.26, 3.00). The duration from the onset to the acquisition of EP results was 1.17 days (0.92, 3.00) (Table 2).

**Table 2:** The temporal profiles of acute spinal cord infarction.

	**Value**
**Time to nadir**	***n*** **(%)**
<6 h	9 (64.3)
6 to <24 h	4 (28.6)
24 to <72 h	1 (7.1)
≥72 h	0
Form of visit	***n*** **(%)**
Emergency center	10 (71.4)
Outpatient clinic	3 (21.4)
From another hospital	0
In–hospital onset	1 (7.1)
	**Median (IQR)**
Onset to hospital (d)	0.17 (0.1, 1)
Onset to T2 image (d)	0.33 (0.14, 1)
Onset to DWI (d)	2.75 (1.26, 3)
Onset to EDX (d) (*n* = 10)	1.17 (0.92, 3)

*h, hours; IQR, interquartile range; d, days; DWI, diffusion weighted imaging; EDX, electrodiagnosis*.

On the other hand, the TM group showed significant differences compared to the SCI group, especially in terms of the temporal profile. In 13 patients (86.7%), it took more than 72 h to the nadir deficit. It took 5 days (3, 7) from the onset to the first hospital visit, and 10 patients (66.7%) visited the hospital out-patient clinic. Regarding the initial serum inflammatory indexes, there were no significant differences in ESR and WBC counts between the two groups. Meanwhile, CRP values were significantly higher in the TM group−0.08 (0.07, 0.13) in the SCI group and 0.21 (0.10, 0.59) in the TM group (*P* = 0.017) (Supplementary Table 1). All 14 SCI patients showed negative inflammatory findings in the CSF study.

### MRI Findings

**Figure 1:**
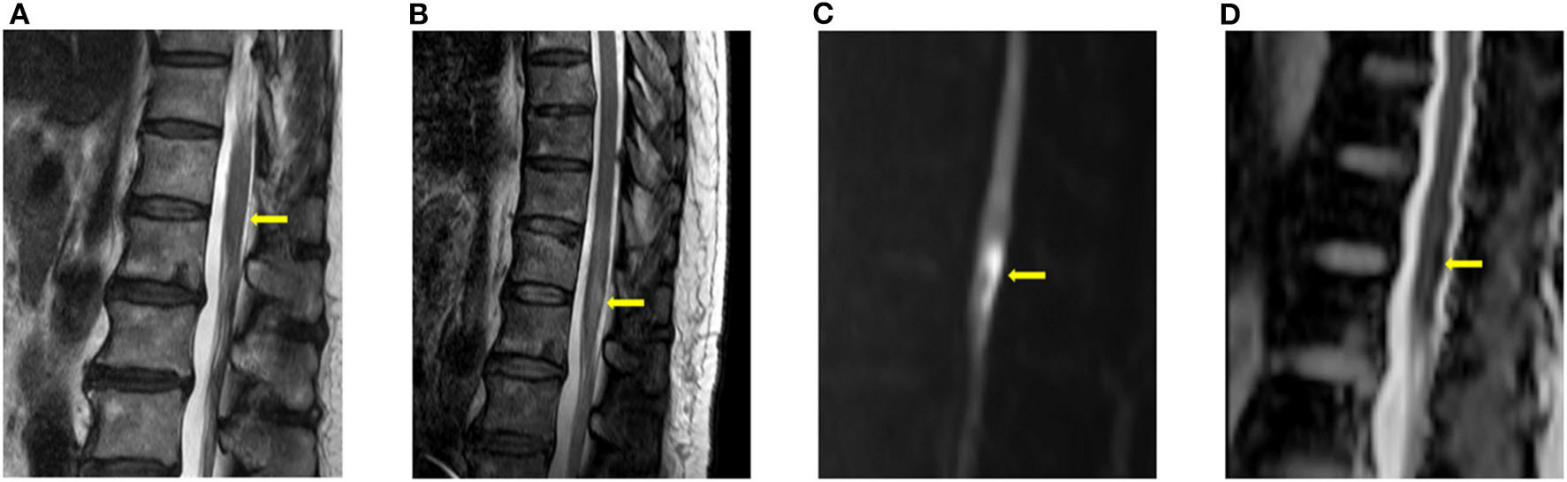
Typical imaging appearances of acute spinal cord infarction. These were images of a patient with conus medullaris infarction (case 4). **(A)** T2 image obtained 3 h after the onset of symptoms showed no high signal intensity around the conus level. **(B)** Follow-up T2 image obtained on the fourth day after the onset of symptoms showed high signal intensity around the conus level. Diffusion restriction was also observed in the diffusion weighted imaging **(C)** and the apparent diffusion coefficient **(D)** study.

**Figure 2:**
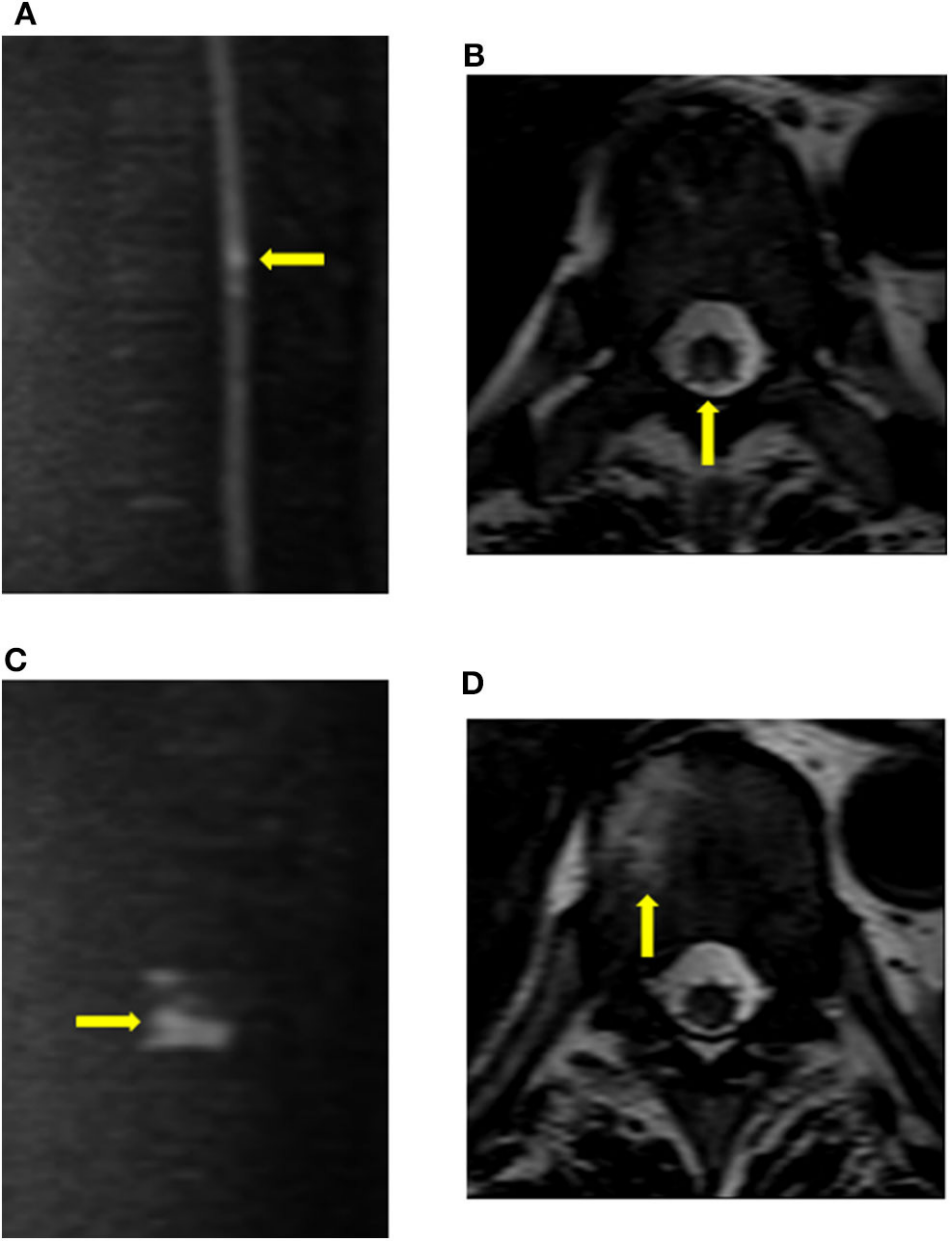
Findings of vertebral body infarction. The patient (case 9) underwent diffusion imaging on the fourth day after the onset of symptoms. **(A,B)** Diffusion restriction was mainly observed in the posterior column of the T9 cord level. **(C,D)** Abnormal signals, which were consistent with vertebral body infarction, were observed within the right vertebral body of T10.

**Figure 3:**
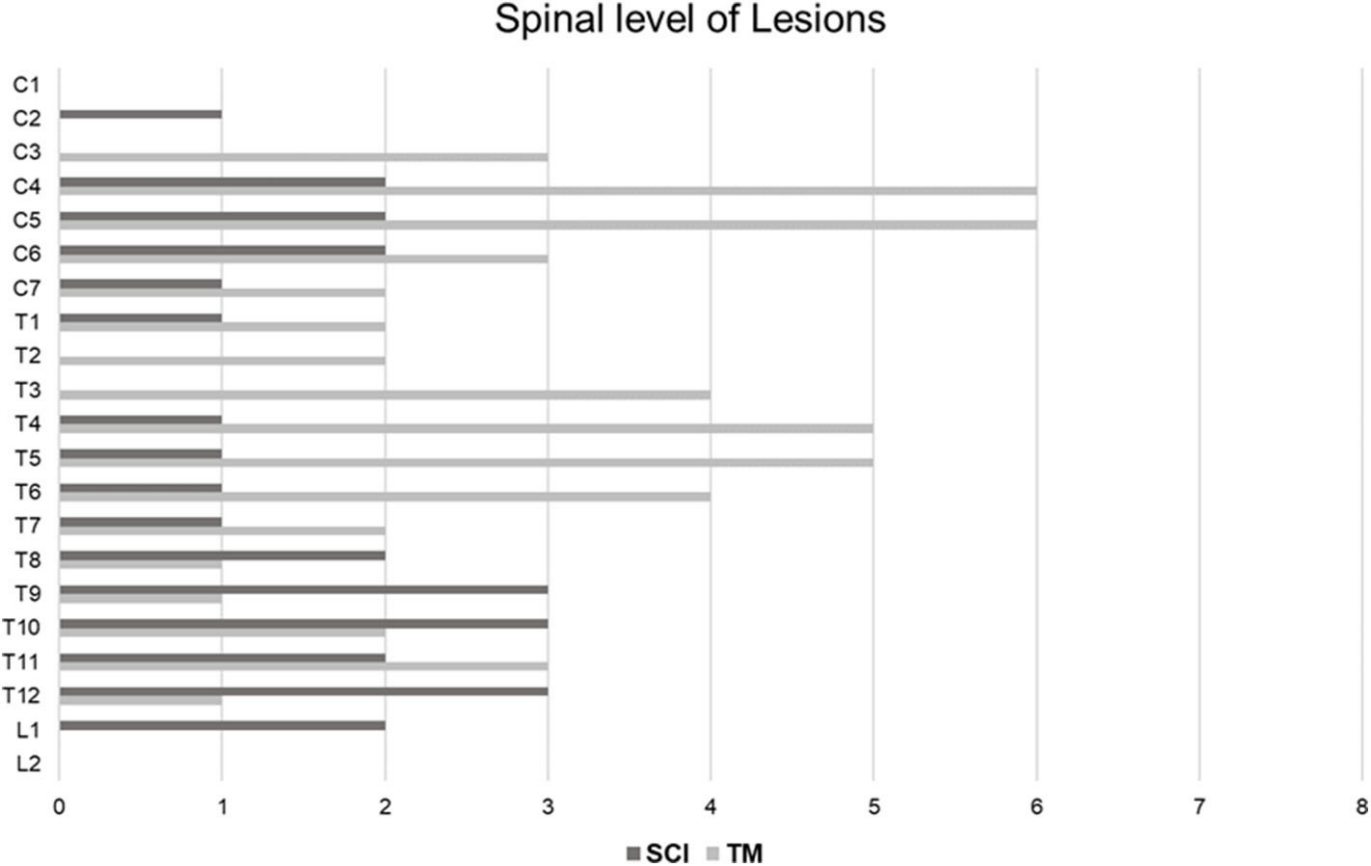
Distribution of SCI and TM at the spinal levels. In the SCI group, a total of 28 spinal levels of all 14 patients were involved. On the other hand, a total of 52 spinal levels of all 15 patients were involved in the TM group. Therefore, the lesion length of the SCI group was shorter than that of the TM group (*P* = 0.005). SCI occurred most frequently in the lower thoracic level, followed by the mid-cervical level. TM was mainly distributed across the cervical and upper thoracic levels. SCI, spinal cord infarction; TM, transverse myelitis.

Table 3 shows the summary of the imaging characteristics of the SCI patients. HSI was present in the initial T2 images of 5 patients (35.7%). For these patients, their T2 images were taken 24 h or more after the onset. Nine patients (64.3%) did not have HSI in their initial T2 images; the T2 images of these patients were taken within 12 h from the onset. Gadolinium enhancement was not observed in any of the patients. All patients showed diffusion restriction in the DWI/ADC study (Figure 1). In our SCI patient group, the earliest time for a positive finding from the diffusion MRI study was 11 h from the onset (case 10). Vertebral body infarction was observed in 5 patients (35.7%) (Figure 2). Regarding the involved spinal levels, the lower thoracic level (T7–12) was the most invaded level, followed by the cervical level. The median value for the length of the invaded level in SCI group was 2 levels (1, 2). Meanwhile, the lesion length in the TM group was 3 levels (2, 4), which was significantly longer than that in the SCI group (*P* < 0.001) (Figure 3). Adjacent vascular evaluations for determining infarction etiology revealed aortic atherosclerosis with calcification in 2 cases and intercostal artery occlusion in 1 case. However, most patients had no accompanying vascular lesions.

**Table 3:** MRI timings and findings for patients with acute spinal cord infarction.

**Case**	**Spinal level**	**Onset to initial T2**	**HSI on initial T2**	**HSI on fu T2**	**HSI patterns**	**Onset to DWI**	**DWI/ADC**	**VB infarction**	**Etiology**
1	C2	3 d	Yes	No fu	Anterior U/V	3 d	Restriction	No	Unknown
2	C4-5	12 h	No	Yes	Anteromedial spot	41 h	Restriction	No	Unknown
3	C4	24 h	Yes	Prominent	Owl's eye	32 h	Restriction	Yes	Unknown
4	T12	3 h	No	Yes	Hologrey	4 d	Restriction	No	Unknown
5	C5-6	4 h	No	Yes	Anterior pencil-like	25 h	Restriction	No	Unknown
6	T6-9	7 h	No	Yes	Holocord	7 d	Restriction	No	Aortic atherosclerosis
7	C6-T1	3 h	No	Yes	Anterior pencil-like and U/V shape	14 h	Restriction	No	Unknown
8	T10	24 h	Yes	Prominent	Hologrey	4 d	Restriction	Yes	Insufficient study
9	T9-10	4 d	Yes	No fu	Posteromedial spot	4 d	Restriction	Yes	Intercostal artery occlusion
10	T4-5	3 h	No	Yes	Owl's eye	11 h	Restriction	No	Unknown
11	T10-11	7 h	No	Yes	Anteromedial spot	48 h	Restriction	No	Unknown
12	T8-9	9 h	No	Yes	Hologrey	3 d	Restriction	No	Aortic atherosclerosis
13	T11-L1	4 h	No	Yes	Hologrey	60 h	Restriction	Yes	Unknown
14	T12-L1	24 h	Yes	Prominent	Anterior pencil-like	7 d	Restriction	Yes	Insufficient study

*HSI, high signal intensity; fu, follow up; DWI, diffusion weighted imaging; ADC, apparent diffusion coefficient; VB, vertebral body; d, days; h, hours*.

### Evoked Potentials

Table 4 summarizes the EP results of all patients. Initial electrodiagnostic data including EP, NCS, and EMG values were available for 10 patients. All of them showed one or more EP abnormalities, such as delayed values or no EP response.

**Table 4:** Values of evoked potentials.

				**Evoked potentials (ms)**				
	**Case**	**Time to EP (d)**	**Time to DWI (d)**	**APB CMCT**	**Median SEP**	**TA CMCT**	**Tibial SEP**	**Pudendal SEP**
SCI	1	3	3	**11.6↑**	18			
(*n* = 10)	2	1.58	1.71	**16.4↑**	**20.8↑**	**NR**	36.1	
	3	1.21	1.33				**42.2↑**	
	4	0.67	4			**NR**	**41.4↑**	39.4
	5	1.13	1.04	**9.7↑**	**21.9↑**	**NR**	**46.1↑**	
	6	10	7			**NR**	**NR**	
	8	1	4			**NR**	**56.3↑**	
	10	1	0.46			**NR**	**41.6↑**	
	12	3	3			**20.8** **↑**	**44.5↑**	37.3
	13	0.17	2.5				**45↑**	37.8
TM	1	30		**12.2↑**	**20.9↑**			
(*n* = 10)	3	15				**20.3↑**	**42.7↑**	
	6	28		**20.5↑**	19.8		34.2	
	7	3					37.0	
	8	11					**42.5↑**	
	9	9				16.3	**43↑**	
	10	14				**29.1↑**	39.2	
	12	8		**13↑**	**21.4↑**	17.1	**41.9↑**	
	13	6				**21.7↑**	**44.7↑**	
	14	4			18.8		**43.3↑**	

*d, days; EP, evoked potential; DWI, diffusion weighted imaging; APB, abductor pollicis brevis; CMCT, central motor conduction time; SEP, somatosensory evoked potential; TA, tibialis anterior; SCI, spinal cord infarction; TM, transverse myelitis; NR, no response; ↑, prolonged value. Bold values mean that they are abnormal*.

All 8 patients who were measured for CMCT had abnormal results. The APB-CMCT results of 3 patients were available, and all of them had prolonged APB-CMCTs. The TA-CMCT results of 7 patients were available; among them, 1 patient had a prolonged TA-CMCT, and 6 patients showed no tibial MEP response. SEP was measured in 10 patients, and 8 of them showed prolonged latencies. The median SEP of case 1 was within the normal range, but her APB-CMCT was delayed. Case 6 had no response for both TA-CMCT and tibial SEP measured on the tenth day from the onset. Three patients underwent the pudendal SEP study, and all of them had values within the normal range of latency.

Regarding the time frames, 7 patients completed the EP study within 48 h from the onset; all of them had abnormal findings. For 5 patients, electrodiagnostic studies were completed prior to the diffusion MRI study, and all of them showed pronounced EP abnormalities. Among them, 3 patients showed both prolonged SEP latency and no TA-MEP responses (case 2, 4, 8). For case 4, TA-MEP was not evoked despite the examination being done just 16 h after the onset. The patient had no peroneal nerve lesion or any L5 root lesion confirmed with NCS and EMG. For 3 patients, though their EP findings were obtained after DWI, both studies were conducted the same day and within a short period. Among these patients, 2 showed both CMCT and SEP abnormalities (case 5, 12). For case 13, the electrodiagnostic test was performed only 4 h after the onset. Out of all the patients, the patient in this case had the shortest duration before the EP study was performed, and the results showed prolonged tibial SEP latency.

On the other hand, for TM patients, it took more than 10 days (5.50, 18.25) from the onset to perform the electrodiagnostic testing; this duration was longer than that of SCI patients. Time of testing also varied widely from patient to patient. We were able to obtain routine NCS, EMG, and EP results for 10 TM patients. Among them, 9 patients had delayed EP values. Abnormalities in both CMCT and SEP were observed in 4 patients. Two patients showed abnormalities only in CMCT. There were no cases in which the EP was not evoked. We also compared the tibial SEP latency between SCI and TM patients. Data for 8 SCI patients and 9 TM patients were available for the analysis. The median value in the SCI group and the TM group was 43.35 ms (41.45, 45.83) and 42.50 ms (38.10, 43.15), respectively. Thus, tibial SEP latency in the SCI group was slightly more prolonged than that of the TM group; however, the difference was not statistically significant (*P* = 0.370).

## Discussion

A distinctive feature of the present study is that it suggests the possibility of using EP study as an additional diagnostic tool for identifying acute SCI. Our results indicate that the immediate sensory and motor EP test can be a useful diagnostic tool for identifying SCI in its early stage. We also aimed to elucidate the usefulness of EP by comparing the EP results of TM patients with those of SCI patients.

By assessing the findings of SCI patients who underwent EP studies within a relatively short time from the onset of symptoms, we observed the following: (1) some MEPs were not evoked; (2) definite abnormalities were noted in both CMCT and SEP in many cases. These findings indicate that a spinal cord lesion triggered by SCI could be detected in a relatively short time using EP testing (17). Five patients even showed clearly abnormal EP results after electrodiagnostic testing, which was done prior to the diffusion MRI study. Although the electrodiagnostic study was done after DWI, 3 patients underwent both tests on the same day, and they too had abnormal EP findings. This means that the likelihood of SCI is higher if, in addition to clinical suspicion, profound EP abnormalities are observed in early electrodiagnostic testing. With an EP study, clinicians can provide an additional basis for conducting a diffusion MRI study for patients suspected to have SCI. Consequently, delays in both diagnosis and misdiagnosis can possibly be reduced.

In the TM group, most patients presented with delays in EP; however, there were no cases in which EP was not evoked. Although the duration from the onset to the completion of testing was shorter for SCI patients than for TM patients, the EP abnormalities of SCI patients were more severe than those of TM patients. Therefore, the early EP findings were also useful for differential diagnosis.

In previous animal studies, histologic changes were generally observed 48 h after induction of cord infarction. Profound gray matter (GM) and white matter (WM) injuries accompanied by neuronal cell damage and a decline in cell numbers were observed, and the boundary between the two areas also became steadily unclear (24, 25). The abnormal spinal cord conduction velocity findings may be primarily related to WM injuries accompanied by myelinated axonal damage, which is known to be eventually correlated with the extent of GM injuries (26, 27). Ultimately, such findings indicate that CMCT could be adopted for the early detection of not only WM injuries but also GM lesions of the spinal cord, which is relatively more sensitive to ischemia. In addition, previous studies have reported that CMCT abnormalities could reflect early microscopic ischemic changes that are not identified by MRI (14, 28). Experimental studies have also demonstrated that MEP responses to ischemia are sensitive and immediate. Tsuda et al. (25) induced spinal infarction in dogs using aortic balloon occlusion and checked for changes in MEP. They discovered that transcranial MEP amplitude reduced by 50% in about 6 min, and completely disappeared in around 7 min. Moreover, apart from MEP providing real-time feedback, it has already been revealed in animal and clinical studies that these changes are correlated with functional outcomes (29–31). Therefore, MEP study can be viewed as a significantly useful tool for the immediate diagnosis of spinal cord injuries.

SEP response to ischemic damage has been shown to be less sensitive than that of MEP, and it is also affected by other factors such as hypotension and low temperature (15, 32, 33). Therefore, it has been often viewed as being less useful than MEP. Additionally, one study reported that the correlation of SEP with motor function was poor, because it mainly reflects the function of the sensory pathway of the posterior column (34). However, from an anatomical perspective, the ascending sensory pathway of the spinal cord is adjacent to the pyramidal tracts. Moreover, other studies have demonstrated that SEP is correlated with motor outcomes related to ischemic injury (35). This is also supported by the distribution of the vascular supply of the spinal cord. The anterior and lateral regions of the spinal cord are mainly supplied by the central and radicular arteries derived from the anterior spinal artery. Meanwhile, the posterior spinal arteries (PSAs) mainly supply not only the posterior column but also the posterior part of the lateral column where the descending motor tracts pass through (36). In addition, the distributions of vascular territories partially overlap, and approximately one third of the entire transection area of the spinal cord is considered to be an overlapping area (37). Therefore, an adjacent motor tract lesion may be identified based on SEP abnormalities. In cases of SCI with pure PSA infarction accompanied by severe sensory symptoms, abnormalities may be observed only in the SEP. Therefore, it is critical to perform both SEP and MEP studies at the same time during the initial assessment of SCI.

As has been described thus far, since MEP and SEP responses to ischemia and reperfusion are rapid, both tests have been applied as key modalities of intraoperative neurophysiological monitoring (IONM) during spinal surgeries (15, 32). IONM requires neural insults of the spinal cord to be assessed in real-time; therefore, complementary MEP and SEP monitoring is suitable for this. Regarding the early diagnosis of SCI, performing sensory and motor EP studies prior to checking for imaging changes in the spinal cord has the advantage of not only having a diagnostic role, but of also being a useful way to assess the extent of spinal cord injury.

For early diagnosis of SCI using EP, it is also important to perform the electrodiagnostic test quickly and report the results immediately. With regard to the time of onset to hospital visit and onset to initial T2 imaging, it was indicated that an MRI was primarily considered in most patients who visited with acute spinal cord syndrome. In our institute, for patients with symptoms of acute spinal cord syndrome, electrodiagnostic tests are generally requested after MRI for verifying neurophysiologic integrity and differential diagnosis. Based on our results, the time difference between the initial MRI and the EP test was within 24 h. Moreover, it can be considered that EP study is highly useful, because it is relatively easy and quick to perform.

In this study, we also described the clinical features and imaging findings of SCI patients. Similar to the findings of previous studies, the SCI patients in this study presented with acute clinical symptoms that worsened rapidly (20, 37). Of the 14 patients in our study, 9 had a rapid progression time of <6 h. A previous report suggests that, in order to diagnose patients with SCI, the duration from the onset of symptoms to nadir should be <72 h (10). All patients in the present study showed a nadir deficit within 72 h from the onset.

SCI occurs most frequently in cases of mid to lower thoracic lesions, and 2 patients in our study developed infarction in their conus medullaris. This area is supplied by the artery of Adamkiewicz and is vulnerable not only to ischemia caused by aortic lesions but also to spontaneous infarctions. It has been known to have a higher metabolic demand than other spinal levels, and thus is more susceptible to ischemia (38–40). Previous studies have also reported that SCI is mainly distributed across the lower thoracic, cervical, and conus levels (8). Thus, the results of the SCI group in the present study are in line with the findings of previous studies. TM on the other hand, is mainly distributed across the cervical and upper thoracic levels. Therefore, the distribution pattern has been emphasized as a feature that could help distinguish SCI from acute inflammatory myelitis, which has a longer lesion length than SCI (5).

In the present study, peri-onset focal adjacent pain was observed in 64.3% of the patients. This rate was similar to or slightly higher than those reported in previous studies (5, 41). Focal adjacent pain is mainly caused by infarction of the associated nerve root or plexus; other causes are vertebral body or meningeal ischemia (42, 43). Mostly, focal adjacent pain manifests as radicular pain around the involved spinal level. In the present study, patients who had thoracic level infarction complained of vague abdominal pain or chest pain. Due to these anatomical concerns, focal pain was not necessarily consistent with the abnormalities of median or tibial SEP in our patient group. Though more than half of the patients with SCI had focal adjacent pain, it was also accompanied with not only inflammatory myelitis, but degenerative spinal disease, arthritis or underlying peripheral nerve diseases (38). Thus, caution needs to be taken before reaching a definite diagnosis.

All our SCI patients showed diffusion restriction in the DWI/ADC study. The majority of the initial T2 studies were performed within 12 h of onset, and all the initial T2 images showed no HSI. Since all patients showed diffusion restriction in the subsequent diffusion MRI study, it can be concluded that it is not easy to diagnose SCI based on an initial T2 image. Vertebral body infarction is known as a confirmatory sign of SCI, and it was observed in 35.7% of the patients in the present study. This rate was similar to those of previous reports (5, 44).

In the present study, 2 patients received delayed diagnoses. In case 1, the abrupt onset of symptoms started with right arm weakness; however, the patient had chronic pain in the right shoulder and mistakenly attributed the weakness of the right arm to the aggravation of a shoulder lesion. Thus, the patient did not visit the hospital on time; the hospital visit was made 3 days after the onset of symptoms. We diagnosed the patient with SCI after conducting T2 MRI and DWI/ADC study at the same time. In case 9, the patient had a chronic right multilevel herniated lumbar disc. At the time of the visit, the only complaint was of radiating pain, but subtle dorsiflexor weakness of the right ankle developed on the second day of admission. We diagnosed the patient with exacerbation of L5 root lesion after performing NCS and EMG; thereafter, we proceeded with a diskectomy, but the patient did not show any subsequent signs of recovery. On the fourth day of admission, the patient suddenly complained of paresthesia, voiding difficulty, hip flexion, and abduction weakness that was rapidly progressing on both sides. We subsequently performed MRI and a DWI/ADC study and diagnosed the patient with SCI at the T9-10 level. CSF analysis conducted immediately after the MRI also presented no evidence of inflammatory changes. This patient experienced focal adjacent pain first, and then focal weakness for a while, after which there was a sudden progression to severe neurologic symptoms. On the MRI performed after the onset of nadir symptoms, we discovered diffusion restriction at the lower thoracic cord level, accompanied by vertebral body infarction.

A proven effective acute treatment for SCI is yet to be established (45). Antiplatelets and anticoagulants have been reported to be administered, but the focus has been on secondary prevention rather than revascularization (46–48). In many cases, steroids were administered simultaneously. The main reason for the administration of steroids is that it is difficult to completely exclude inflammatory myelitis in the early stages of SCI (10, 49). Unlike the case of cerebral infarctions, an acute phase thrombolytic treatment protocol has not been standardized for SCI. Several case reports and ongoing clinical trials have been released, and a common emphasis in the reports is that acute thrombolytic treatment could be performed in a timely manner only if an accurate diagnosis is made prior to treatment (50–53). Therefore, should a systematic acute phase treatment process be established in the future, the importance of an accurate early diagnosis of SCI will be emphasized even more. Based on our results, an immediate sensory and motor EP study performed together with a review of clinical symptoms can provide clues to facilitate the diagnosis and confirmation of SCI prior to the performance of a diffusion MRI study. Furthermore, an EP study can be a useful base study for well-established therapeutic decisions in the future.

This study has a few limitations. This was a retrospective study based on the experience of a single center, and was conducted with a small sample size. In addition, because our sampling period was over 8 years, not all patients underwent the same standardized examination. Furthermore, as seen in the study results, not all patients underwent EP testing. In each patient group, only 10 patients completed electrodiagnostic tests, including EP. Therefore, we were not able to prove whether the results of the EP test had any significant statistical meaning. In the future, systematic researches should be conducted using well-controlled cohorts.

In conclusion, although SCI is rare and difficult to identify immediately, it can be diagnosed through the review of typical characteristics of clinical features and imaging findings. From our observations, we were also able to identify severe EP abnormalities in SCI patients, even in the hyperacute stage of the disease. Therefore, we suggest that for suspected SCI cases, conducting an immediate sensory and motor EP study in a timely manner can improve diagnostic accuracy and speed.

## Data Availability Statement

The raw data supporting the conclusions of this article will be made available by the authors, without undue reservation.

## Ethics Statement

The studies involving human participants were reviewed and approved by the Institutional Review Board of Pohang Stroke and Spine Hospital reviewed and approved this study (Approval No. PSSH0475-202004-HR005). Written informed consent for participation was not required for this study in accordance with the national legislation and the institutional requirements. Written informed consent was obtained from the individual(s) for the publication of any potentially identifiable images or data included in this article.

## Author Contributions

DP and SEL: writing the manuscript, data collection, and interpretation. BHK, JKP, and JMC: data analysis, diagnosis, and procedures of patients. HDK and SYL: editing the manuscript and supervising the entire study. All authors reviewed the manuscript and were in full agreement to submission.

## Conflict of Interest

The authors declare that the research was conducted in the absence of any commercial or financial relationships that could be construed as a potential conflict of interest.
