# Supplementary material for: Spinal Cord Infarction: A Single Center Experience and the Usefulness of Evoked Potential as an Early Diagnostic Tool
Source: Front Neurol. 2020 Oct 27;11:563553. doi: 10.3389/fneur.2020.563553 (PMC7652817; doi:10.3389/fneur.2020.563553)
Supplement: Supplementary file 1 [file Table_1.DOCX]

**Supplementary table 1.** Comparison of SCI and TM patients

|  | SCI (n = 14) | TM (n = 15) | P value |
| --- | --- | --- | --- |
| Age, yr | 65.5 (58, 71) | 60.0 (57, 65) | 0.377 |
| Sex, male | 8 | 11 | 0.368 |
| Spinal level, n |  |  | 0.005 |
| Cervical | 8 | 20 |  |
| Upper thoracic (T1-T6) | 4 | 22 |  |
| Lower thoracic (T7-T12) | 15 | 14 |  |
| Upper lumbar | 2 | 0 |  |
| Length of level | 2.0 (1, 2) | 3.0 (2, 4) | <0.001 |
| Onset to nadir deficit, n (%) |  |  | <0.001 |
| <6 hr | 9 (64.3) | 0 |  |
| 6 to 24 hr | 4 (28.6) | 0 |  |
| 24 to 72 hr | 1 (7.1) | 2 (13.3) |  |
| >72 hr | 0 | 13 (86.7) |  |
| Form of visit, n (%) |  |  | <0.001 |
| emergency center | 10 (71.4) | 0 |  |
| outpatient clinic | 3 (21.4) | 10 (66.7) |  |
| transferred from other hospital | 0 | 5 (33.3) |  |
| in–hospital onset | 1 (7.1) | 0 |  |
| Onset to hospital, days | 0.17 (0.1, 1) | 5 (3, 7) | <0.001 |
| Onset to T2, days | 0.33 (0.14, 1) | 9 (6, 14) | <0.001 |
| Onset to DWI, days | 2.75 (1.26, 3) | none |  |
| Onset to electrodiagnosis, days | 1.17 (0.92, 3) | 11 (7, 21.5) | <0.001 |
| Tibial SEP latency, ms (n=8:9) | 43.35 (41.45, 45.83) | 42.50 (38.10, 43.15) | 0.370 |
| mRS of nadir deficit | 3 (2, 4) | 1 (1, 2) | 0.003 |
| Laboratory findings (Serum) |  |  |  |
| WBC count, x10^3^/uL | 6700 (5230, 8965) | 8900 (6530, 10970) | 0.058 |
| ESR, mm/hr | 8 (4, 19.5) | 9 (8, 20) | 0.405 |
| CRP, mg/dl | 0.08 (0.07, 0.13) | 0.21 (0.10, 0.59) | 0.017 |

SCI, spinal cord infarction; TM, transverse myelitis; DWI, diffusion weighted imaging; SEP, somatosensory evoked potential; mRS, modified Rankin scale; WBC, white blood cell; ESR, erythrocyte sedimentation rate; CRP, C-reactive protein.
